# Identification of Raf-Like Kinases B Subfamily Genes in *Gossypium* Species Revealed *GhRAF42* Enhanced Salt Tolerance in Cotton

**DOI:** 10.3390/ijms222312649

**Published:** 2021-11-23

**Authors:** Zhen Peng, Xuran Jiang, Zhenzhen Wang, Xiaoyang Wang, Hongge Li, Shoupu He, Zhaoe Pan, Abdul Qayyum, Abdul Rehman, Xiongming Du

**Affiliations:** 1 Zhengzhou Research Base, State Key Laboratory of Cotton Biology, Zhengzhou University, Zhengzhou 450000, China; pengzhen01@caas.cn (Z.P.); jiangxurann@163.com (X.J.); lihongge@caas.cn (H.L.); heshoupu@caas.cn (S.H.); panzhaoe@caas.cn (Z.P.); 2 State Key Laboratory of Cotton Biology, Institute of Cotton Research Chinese Academy of Agricultural Science, Anyang 455000, China; shenhuawzz@163.com (Z.W.); wangxiaoyang198806@126.com (X.W.); 3 National Nanfan Research Institute (Sanya), Chinese Academy of Agricultural Sciences, Sanya 572024, China; 4 Department of Plant Breeding and Genetics, Bahauddin Zakariya University, Multan 66000, Pakistan; raoqayyim@bzu.edu.pk

**Keywords:** Raf-like kinase, synteny, VIGS, gene expression, genome-wide analysis

## Abstract

Salinity is a critical abiotic factor that significantly reduces agricultural production. Cotton is an important fiber crop and a pioneer on saline soil, hence genetic architecture that underpins salt tolerance should be thoroughly investigated. The Raf-like kinase B-subfamily (RAF) genes were discovered to regulate the salt stress response in cotton plants. However, understanding the RAFs in cotton, such as Enhanced Disease Resistance 1 and Constitutive Triple Response 1 kinase, remains a mystery. This study obtained 29, 28, 56, and 54 RAF genes from *G. arboreum*, *G. raimondii, G. hirsutum*, and *G. barbadense,* respectively. The RAF gene family described allopolyploidy and hybridization events in allotetraploid cotton evolutionary connections. Ka/Ks analysis advocates that cotton evolution was subjected to an intense purifying selection of the RAF gene family. Interestingly, integrated analysis of synteny and gene collinearity suggested dispersed and segmental duplication events involved in the extension of RAFs in cotton. Transcriptome studies, functional validation, and virus-induced gene silencing on salt treatments revealed that *GhRAF42* is engaged in salt tolerance in upland cotton. This research might lead to a better understanding of the role of RAFs in plants and the identification of suitable candidate salt-tolerant genes for cotton breeding.

## 1. Introduction

Cotton is a major crop around the globe that might be used as a pioneer crop for saline-alkali soil reclamation, resulting in a more suitable cropland for plant growth [1]. Soil salinity is a key factor that threatens crop productivity, the environment, and agricultural sustainability [2,3]. Indeed, salinity has afflicted almost one billion hectares of dry and semi-arid land worldwide [4]. Soil salinity will rise dramatically over time due to climate change, inappropriate irrigation, and excessive use of fertilizers [5]. By 2050, salt salinity is expected to impact more than half of the arable land [6]. Excessive salt buildup in soil creates a severe menace to agricultural productivity [7,8]. Cotton is a relatively resistant plant to salt stress [9,10]. However, the exact mechanism behind the salt tolerance of cotton is still unknown. 

Cotton salt stress responses have been linked with a variety of components concerning transcriptome profiling. Cotton research has entered a postgenomic age after obtaining genomic sequences of diploid and tetraploid cotton [11,12,13]. Functional genomic techniques for transitory silencing of endogenous genes in cotton have been developed, such as Agrobacterium-mediated virus-induced gene silencing (VIGS) [14,15,16]. These breakthroughs enable us for genome-wide functional study of cotton genes. Cotton zinc finger protein1 (GhZFP1) improves salt tolerance in tobacco [17]. In Arabidopsis, overexpression of calcineurin B-like (CBL) kinase 6 (GhCIPK6) improves salt and drought tolerance [18]. miRNVL5, a cotton microRNA ovule line 5 was implicated in salt stress tolerance [19]. To address the future challenges, plant breeders are striving to improve cotton genotypes for marginal environments against salt.

MAPK (mitogen-activated protein kinase) networks in eukaryotes are ubiquitous signaling components that perform critical functions in stress response and plant growth and development. MAPK kinase kinase (MAPKKK), MAPK kinase (MAPKK), and MAPK are the three kinases that build up the MAPK cascade. The MAPK cascades transmit signals via phosphorylation and activation in a specific order [20]. MAPK networks are well-protected signaling pathways in all eukaryotes that are frequently engaged in plant response mechanisms [21]. Different cellular functions can be triggered by MAPK cascades such as respond to environmental stresses and predetermined cell death [22,23]. Upstream phosphorylation components are responsible for the activation of kinases [24]. The Arabidopsis genome contains around 10 MAPKKs, 20 MAPKs, and 80 MAPKKKs, and splits MAPKKKs into two subtypes, Raf-like kinases and MEKK-like [20,25]. MAPKKKs have been characterized as Raf-like protein kinases (RAFs) in plants [20,26]. At the same time, the Raf-like kinase family contains 48 members in Arabidopsis. Raf-like protein kinases are divided into four B and seven C subgroups [20]. B1 subgroup contains four members, B2 and B3 comprise on six members, and B4 have seven members [27]

In the *Arabidopsis thaliana*, Raf-like Kinase, Enhanced Disease Resistance 1 (EDR1) and Constitutive Triple Response 1 (CTR1), a B3 subfamily of Raf-like kinase played a vital role in ethylene and disease resistance signaling [28]. RAF2 belongs to B3 family, also known as EDR1, suppresses the immunological response to biotic stress [29]. The serine/threonine protein kinase CTR1 functions as a critical negative component in the ethylene signaling pathway in Arabidopsis [30]. Likewise, mutants of many Raf-like protein kinases affiliated with B2 and B3 families [31,32], are hypersensitive to salt stress and insensitive to ABA2 [33]. Based on amino acid sequences, Raf10 is categorized as a MAPKKKs [32]. Raf10 and its homolog Raf11 are key ABA response regulators which involved in seed dormancy and seedling ABA sensitivity [32]. Raf-like MAP3Ks are thought to play essential functions in plant development and are also involved in signal transduction pathways between plant and hormones in diverse environmental stresses [34,35].

Crop plants have evolved various biochemical and physiological processes to respond salt stress [36]. The stress-determining genes, control the downstream gene’s expression, activate transcription factors, and increase stress tolerance [37]. We first identified potential RAF-like kinase genes in four cotton species based on their transcriptome data in the present work. Then we examined the gene’s expression in different tissues at various stress levels to select salt stress tolerance candidate genes. PCR-qRT verified the presence of salt-tolerant gene *GhRAF42* in cotton. The VIGS technology was also utilized to validate the findings. Our results will provide the basic knowledge of the function and evolution of cotton RAF-like kinase and potential genes for developing salt tolerance cotton genotypes.

## 2. Results

### 2.1. RAF Sequence Analysis and Characterization of Four Cotton Species

**Figure 1:**
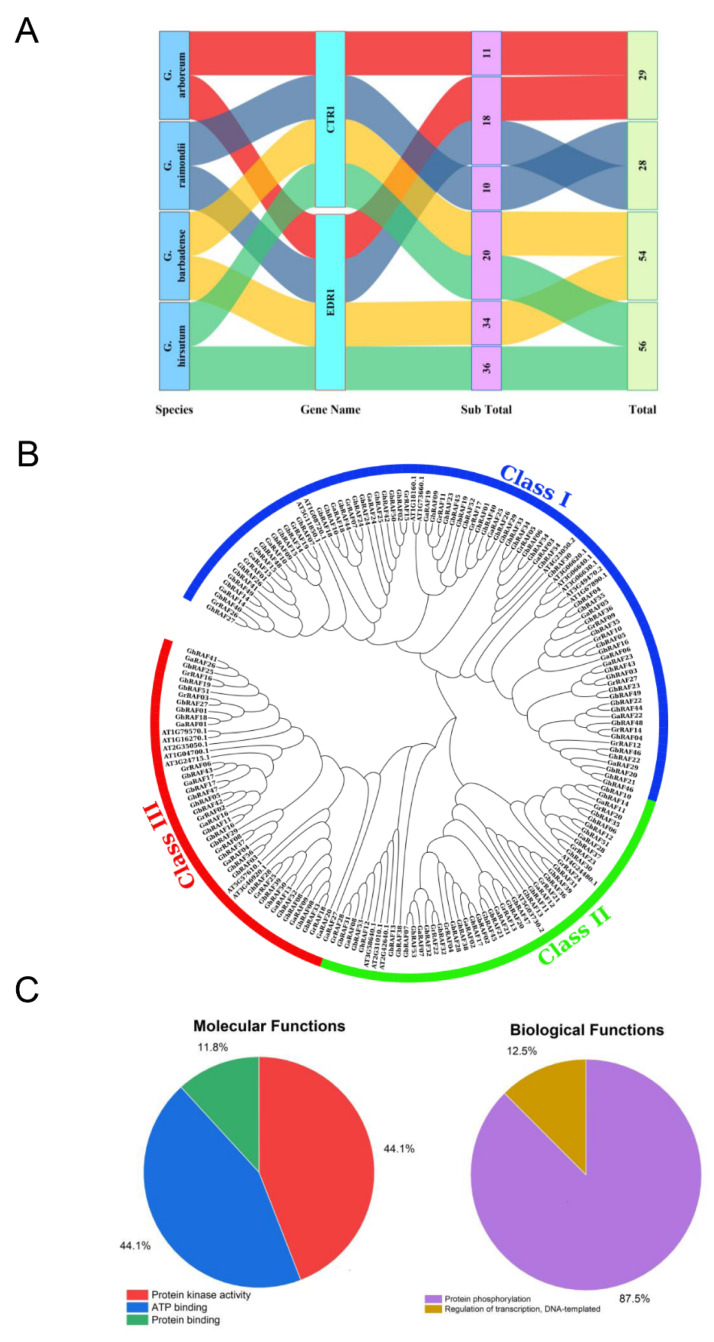
Salient features of The Raf-like kinase B-subfamily (RAF) genes and types, RAF genes phylogenetic analysis in four species of cotton and study of gene ontology (**A**) Description of RAF genes (**B**) Phylogenetic analyses (**C**) Gene ontology enrichment of *GhRAFs*.

Four cotton genomes (two diploids and two tetraploid species) were obtained from CottonFGD database and analyzed using HMMER and BLASTP software to find genes encoding the RAF protein domain. After domain and alignment analysis, a total of 167 RAF proteins were found in the current study (Table S2). *G. raimondii* illustrated 28 RAF-like kinase genes (18 EDR1 and 10 CTR1), while *G. arboreum* exhibited 29 genes (18 EDR1 and 11 CTR1). Additionally, *G. hirsutum* represented 56 genes (36 EDR1 and 20 CTR1), and *G. barbadense* exhibited 54 RAF genes (34 EDR1 and 20 CTR1) (Figure 1A). Candidate RAF genes were renamed based on their chromosomal locations [38]. *G. raimondii* and *G. hirsutum* exhibited 96~1403 amino acids residues, *G. arboreum* contained 119~1401, and *G. barbadense* had 150~1401 amino acids. The BUSCA analyzed the RAF protein sequence and predict that most genes in four cotton species were found in the nucleus (Table S2). *GrRAF01*, *GrRAF07*, *GrRAF19*, and *GrRAF26*, were identified in the chloroplast, *GrRAF11* and *GrRAF17* in the endoplasmic membrane, and *GrRAF02* was found in the plasma membrane. Eight of the twenty-nine genes were found in the chloroplast, endomembrane system, and plasma membrane in *G. arboreum*. Fifteen *GbRAFs* genes were found in the chloroplast, endomembrane system, and plasma membrane. Similarly, only 17 *GhRAFs* genes were found outside the nucleus from 56 genes of *G. hirsutum*.

MEGA X was used to build the phylogenetic tree using multiple sequence alignment of 28 RAF proteins from *G. raimondii*, 29 RAF proteins from *G. arboreum*, 56 from *G. hirsutum*, 54 from *G. barbadense*, and 22 *A. thaliana* proteins (Figure 1B). The phylogenetic tree demonstrated that 189 RAFs genes could be naturally categorized into 3 groups. Class I was observed as the largest, containing 91 proteins (13 *GrRAFs*, 13 *GaRAFs*, 30 *GhRAFs*, 25 *GbRAFs*, and 10 *AthRAFs*). Similarly, 50 RAFs proteins were present in group II (8 *GrRAFs*, 7 *GaRAFs*, 14 *GhRAFs*, 16 *GbRAFs,* and 5 *AthRAFs*), whereas class III exhibited 48 RAFs proteins (7 *GrRAFs*, 9 *GaRAFs*, 12 *GhRAFs*, 13 *GbRAFs,* and 7 *AthRAFs*). Group I had the most members in the RAF gene family, followed by groups II and III. Clustering of GrRAFs within each subgroup with rest all cotton species have a close evolutionary relationship, hence providing evidence supporting the origin of tetraploid species from the hybridization of A and D genomes [11].

The molecular and biological functions of four cotton species were retrieved from GO ontology from the CottonFGD database (Figure 1C). Molecular functions of RAF genes of *G. hirsutum* revealed that functions of both protein kinase activity and ATP binding was 44.1% and function of protein binding was 11.8%. Biological functions involved in the regulation of transcription and DNA template contribute 87.5%, and protein phosphorylation contributed 12.5%. Gene ontology of *G. arboreum*, *G. raimondii,* and *G. barbadense* is illustrated in Figure S1.

### 2.2. Cotton RAF Gene Family Tree Diagram, Exon-Intron Structure, Motif Assay, and Gene Ontology Analysis

**Figure 2:**
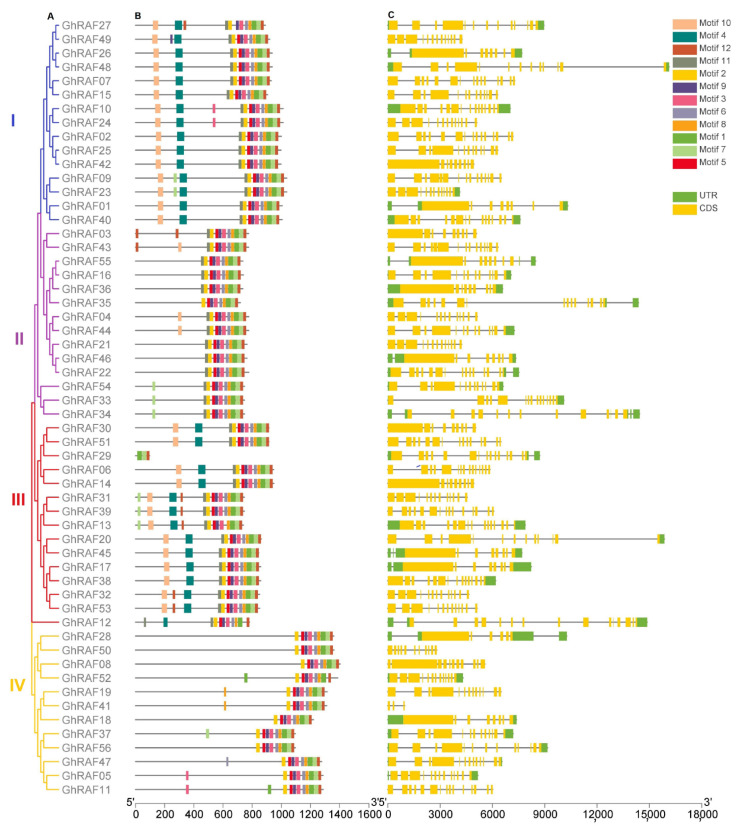
Phylogenetic relationship, motif, and gene structures of RAF gene members in *G. hirsutum* (**A**) Evolutionary relationship (**B**) Structure of conserved motifs (**C**) Gene architecture.

Phylogenetic tree, gene structure, and conserved domains were evaluated to describe the potential correlation between the evolutionary process and gene function/gene structure of cotton RAF genes. MEGA X was used to illustrate the evolutionary tree with the help of neighbor-joining method [39]. All positions with a site coverage of less than 90% were removed. The resulting dataset had a complete set of 296 positions in the composite tree of *A. thaliana* and four cotton species. Individual trees of *G. raimondii*, *G. arboreum*, *G. hirsutum*, *G. barbadense* had 87, 94, 77, and 76 positions, respectively, in the final dataset with complete deletion. In the RAF gene family, *G. barbadense* exhibited maximum exons (18), followed by *G. raimondii* (16), *G. hirsutum* (15), and *G. arboreum* (15) (Figure 2 and Figure S2). *GhRAF17* (5649 bp) was the longest gene, followed by *GbRAF51* (5623 bp), *GrRAF18* (5275 bp), and *GaRAF09* (4206 bp) (Figure 2 and Figure S2). Introns and exons were arranged to reveal the evolutionary connections between various gene families. There was also an important link between exon-intron structure and phylogeny. MEME was used for the coding of the motif structure of all the RAF protein sequences of cotton. In cotton RAFs proteins, 12 motifs were detected; intriguingly, motifs 2 were found to be conserved in all RAF proteins sequences in all-cotton species. Specifically, in *G. hirsutum,* all the group proteins contain motifs 1, 2, 3, 6, and 7. Likewise, motif 5 is present in all proteins except *GhRAF27* and *GhRAF29*. Moreover, motifs 8 and 9 were present in all proteins except *GhRAF29*. Most conserved domain with all 12 conserved motifs were found in Group I. The closely related genes exhibited typical motif composition and were more structurally similar with exon and intron lengths that differ significantly. These results revealed functional similarity between proteins of GhRAFs in paralogous pairs. Information about motif logo, motif e-value, motif sites, motif width, motif conserved amino acids, and motif similarity matrix of four cotton species are provided in Table S3.

### 2.3. Chromosomal Gene Location and Synteny Analysis 

**Figure 3:**
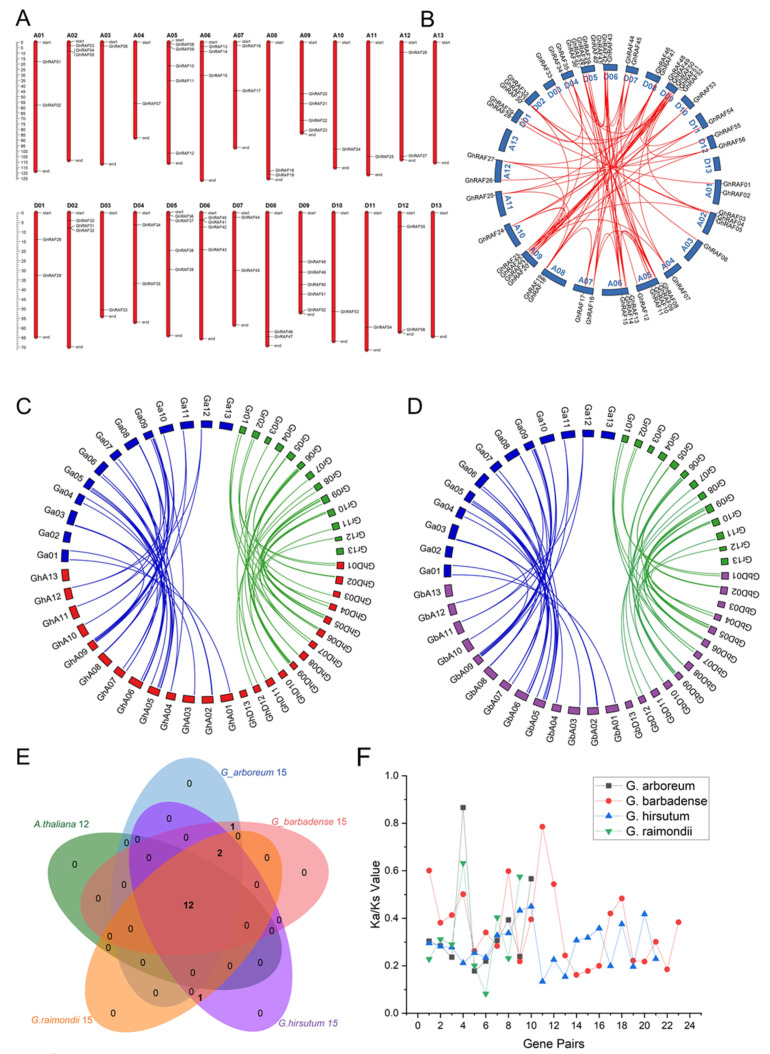
RAF gene distribution on chromosomes, collinearity, synteny and duplication events analysis of RAF genes in cotton (**A**) Chromosomal map (**B**) Circos diagram of *G. hirsutum* (**C**) Synteny diagram involving *G. arboreum*, *G. raimondii* and *G. hirsutum* (**D**) Synteny diagram involving *G. arboreum*, *G. raimondii* and *G. barbadense* (**E**) Venn Diagram among different species clusters (**F**) Synonymous and nonsynonymous ratio.

Mapchart assisted in the designing and exact localization of genes on chromosomes. It was observed that each chromosome carried a various number of RAF genes in each species of cotton. For example, D09 chromosome in *G. raimondii* exhibited the highest number of genes. Likewise, the A05 and A09 chromosomes in *G. arboreum* had the most RAF genes, followed by *G. hirsutum* on A09 and D09, and *G. barbadense* on A05, A09, D05, D06, and D09. The longest chromosomes were D13 (85.32 Mb), A03 (135.70 Mb), A06 (126.48 Mb), and A08 (119.88 Mb) in *G. raimondii*, *G. arboreum*, *G. hirsutum*, and *G. barbadense,* respectively (Figure 3A and Figure S3).

Gene duplication is a common cause of gene expansion and the development of novel gene functions. In the present study, TBtools performed an amino acid homologous blast of four cotton genomes. In *G. raimondii*, 12,949, 707, and 1897 collinear genes, collinear blocks, and tandem repeat genes were noticed, respectively (Table S4). In *G. arboreum*, 2375 tandem repeat genes, 1022 collinear blocks, and 15,891 collinear genes were observed. Likewise, *G. hirsutum* contains 55,604 collinear genes, 2225 collinear blocks, and 3334 tandem repeat genes in its genome. In *G. barbadense*, 56,139 collinear genes, 2215 collinear blocks, and 3304 tandem repeat genes were discovered (Table S4). Paralogous pairings of RAF gene families arose through whole genome duplications or segmental duplications. Transpositions involving only a tiny number of tandem repeat genes might result in a high number of members in four cotton species. In *G. raimondii*, scattered, segmental, and proximal duplications were found in 13, 14, and 01 genes, respectively. In *G. arboreum*, there were ten scattered, eighteen segmental, and one tandem and proximal duplications (Table S5). In tetraploid cotton species, *G. hirsutum* had fifty-four segmental and one dispersed duplication, whereas *G. barbadense* had six scattered, forty-seven segmental, and one tandem gene duplication (Table S5). Hence, it could be concluded that segmental duplications played a vital role in the evolution of RAF-like kinase gene family in cotton. In the synteny diagram using circos, red lines depicted gene duplications between chromosomes within genomes. Cotton RAF genes were non-randomly distributed with most homologous genes evenly split between the A and D genomes. *G. hirsutum* exhibited 77 gene duplications within a genome (Figure 3B). The highest gene duplications were illustrated by chromosomes A09 and D09. Interestingly, all the genes demonstrated their duplicated gene copy on the A and D genome except *GhRAF02*. The duplication and divergence of gene members during the development of gene families leads to functional diversity in the biological processes carried out by the gene family. Similar information about *G. raimondii*, *G. arboreum*, and *G. barbadense* is described in Figure S4. Synteny was drawn between D-Gh Dt, D-Gb Dt, A-Gh At gene pairs from *G. raimondii* to further explore the evolution and origin of cotton RAF genes of *G. arboreum*, *G. hirsutum* and *G. barbadense* (Figure 3C,D). Synteny diagram was plotted between A genome of *G. arboreum* with A genome of *G.* hirsutum and D genome of *G. raimondii* with D genome of *G. hirsutum* and similar pattern with *G. barbadense.* As expected, most cotton RAF genes A genome of *G. arboreum*, corresponded to A genome of *G. hirsutum*, and similarly D genome of *G. raimondii*, compared to D genome of *G. hirsutum*. For example, chromosome Ga05 and Ga09 in *G. arboreum* was syntenic to the region GhA05 and GhA09 in *G. hirsutum,* respectively. All the chromosomes of both species have orthologs on each chromosome except Ga13 and GhA13. Remarkably, similar behavior was recorded between *G. raimondii* D genome and *G. hirsutum* D genome chromosomes. GrD06 and GrD09 chromosomes of *G. raimondii* and GhD05 and GhD09 of *G. hirsutum* exhibited the highest duplicated genes. Almost the same syntenic relationship was also observed between diploid A and D genome with A and D genome of tetraploid *G. barbadense* (Figure S3). These findings represented that duplication of cotton RAF genes during evolution might play a key role in cotton growth and defensive response as well as enhances our understanding of the functional diversity of cotton RAF genes.

### 2.4. Orthologous Gene Clusters Identification and Synonymous and Nonsynonymous Ratio

Orthologous RAF gene clusters in *A. thaliana*, *G. raimondii*, *G. arboreum*, *G. hirsutum*, and *G. barbadense* were assessed to investigate the event of polyploidization throughout the evolutionary phase of the RAF gene family in cotton A and D genomes. The identified clusters of orthologous genes and their overlap positions are depicted in Figure 3E. Surprisingly, the magnitude of the contribution of each species of cotton was found to be identical. The discovery of 12 RAF orthologous gene clusters in cotton suggested that polyploidization has led to developing novel orthologues gene clusters. Similarly, orthologue gene groups were generated in *A. thaliana* and between each cotton species (Table S6). Furthermore, the data revealed that as evolutionary distances increase, the number of known orthologue genes decreases. Comparatively, *G. raimondii*, *G. arboreum*, *G. hirsutum*, *G. barbadense* had 562, 331, 525, and 562 orthologous genes, respectively, whereas *A. thaliana* exhibited 60 orthologous genes (Table S6). In *G. arboreum*, *G. barbadense*, and *A. thaliana* represented 46, 91, and 197 co-orthologs, respectively, whereas *G. hirsutum* and *G. raimondii* illustrated 107 co-orthologs. Intriguingly, two singletons were found in *G. barbadense*, while one singleton was found in *G. arboreum*. 176 in-paralogs were identified in *G. raimondii* and *G. hirsutum*, whereas 26 and 138 in-paralogs were found in *G. arboreum* and *G. barbadense*, respectively. 

The Ka/Ks ratio estimates the evolutionary history of a gene region or gene [40]. 9, 10, 21 and 23 gene duplication pairs were found in *G. raimondii*, *G. arboreum*, *G. hirsutum* and *G. barbadense*, respectively (Figure 3F). It was observed in four cotton species that the Ka/Ks ratio for all RAFs genes understudy is less than one suggested purifying selection (Table S7). Segmental duplications of the RAF genes occurred in *G. arboreum* between 1.86 and 47.68 Mya. Similarly, the RAF genes in *G. raimondii* were duplicated between 2.29 and 45.72 Mya. Additionally, segmental duplication occurred between 13.53~142.07 and 0.77~2.09 Mya in *G. hirsutum* and *G. barbadense*, respectively (Table S7). Hence, it could be concluded that A and D genomes are the progenitors of all cotton species.

### 2.5. Screening of Salt Tolerance Genes Based on Transcriptome Data

**Figure 4:**
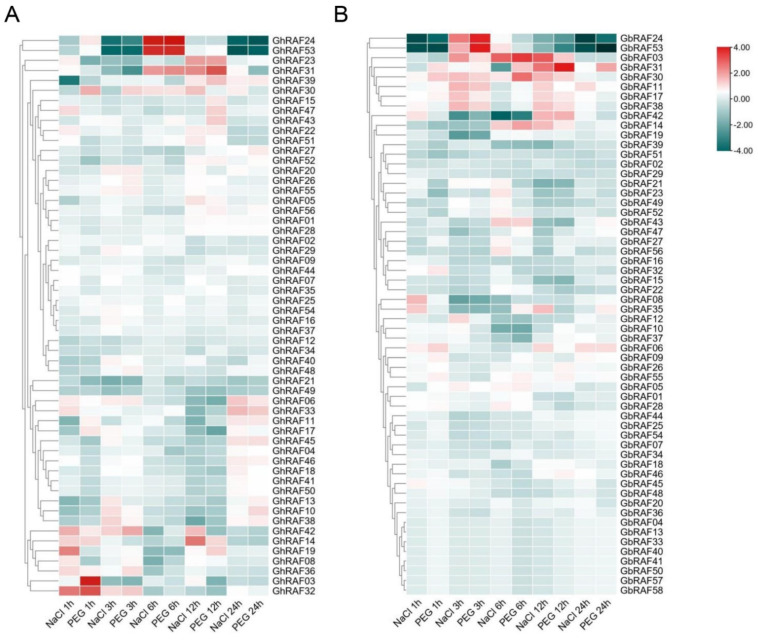
Expression of GhRAF and GbRAF member genes under salt and PEG stress at various intervals of time and acquisition of candidate genes related to salt tolerance (**A**) Transcriptome expression data of *G. hirsutum* (**B**) Transcriptome expression data of *G. barbadense.*

Expression patterns of salt tolerance RAF family members in *G. arboreum*, *G. hirsutum,* and *G. barbadense* were analyzed using published transcriptome databases [13,41,42]. The expression pattern of the RAF gene under polyethylene glycol (PEG) and salt stress was identified at various intervals of time using publicly accessible high throughput RNA seq data to elucidate the potential functional roles of the RAF gene family in cotton. The heatmap of RAF gene expression was designed using TBtools. The expression profile of GhRAF members reveals significant differences (Figure 4A). Across two stress levels at different time intervals, more than half of *GhRAF* genes were expressed at a low level. Approximately 25% of *GhRAF* genes exhibited a broad expression range and consistently high expression. Furthermore, several of them had stress-related expressions. Genes *GhRAF42*, *GhRAF52*, and *GhRAF05*, for example, showed high expression at one stress level but low expression in another, suggesting that these three genes may play critical roles in cotton reproductive biology. Moreover, *GhRAF24*, *GhRAF53*, *GhRAF23*, *GhRAF31, GhRAF42, GhRAF14,* and *GhRAF32* were interested in expression analysis. The function of duplicated genes differed in three primary ways due to gene duplication, which is common in tetraploid cotton genomes: pseudogene copies, homologous genes, and new genes with unique roles [13]. The heat map showed that most homologues in the RAF gene family were functionally conserved, while just a few had distinct gene functions. Surprisingly, *G. barbadense* demonstrated similar behavior concerning expression (Figure 4B). All of the aforementioned findings suggest that the cotton RAF gene family has a lot of functional diversity. 

### 2.6. GhRAF Gene Expression Characteristics Analysis

**Figure 5:**
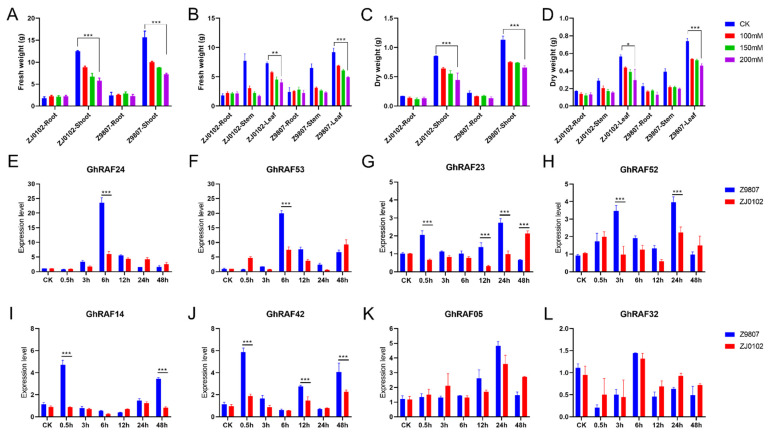
Evaluation of different salt stress treatments on root shoot weight for RT-qPCR and expression analysis of candidate genes (**A**) Fresh weight of root and shoot (**B**) Fresh weight of root, stem and leaf (**C**) Dry weight of root and shoot (**D**) Dry weight of root, stem and leaf. Expression analysis of *GhRAF24* (**E**); GhRAF53 (**F**); *GhRAF23* (**G**); *GhRAF52* (**H**); *GhRAF14* (**I**); *GhRAF42* (**J**); *GhRAF05* (**K**); *GhRAF32* (**L**). CK means NaCl-untreated as control.0.5–48 h means different time points after NaCl stress. The significant difference is Student ’s t test; * *p* < 0.05; ** *p* < 0.01; *** *p* < 0.001.

An experiment was conducted to detect the optimal salt stress concentration in cotton. Both genotypes were grown in the glasshouse under the same pre-requisite conditions as described in Section 2.7. After 100 mM, 150 mM, and 200 mM of salt treatments fresh and dry root, shoot, and leaves were weighed. Fresh shoot weight was substantially lower in both genotypes under 200 mM NaCl treatment than control, but fresh root weight was non-significant (Figure 5A). Fresh leaf weight, dry shoot weight, and dry leaf weight showed similar results (Figure 5B–D). Consequently, it was found that the 200 mM NaCl treatment severely damaged the cotton plants, resulting in lower fresh and dry weight in the root, shoot, and leaves compared to the control and other treatments, i.e., 100 and 150 mM. Hence, 200 mM salt concentration was selected to analyze the candidate gene expression profiles. After extracting high-quality RNA and reverse transcription cDNA from samples at 6 time points after NaCl treated (Figure S5), A RT-qPCR experiment was conducted to validate the expression features of the *GhRAF24*, *GhRAF53*, *GhRAF23*, *GhRAF52*, *GhRAF14*, *GhRAF42*, *GhRAF05*, and *GhRAF32* genes. At 200 mM salt stress, the expression of the genes was differently regulated in the leaves of two cotton genotypes at various time intervals (Figure 5E–L). At 200 mM NaCl concentration, *GhRAF42* gene expression increased in the leaves of Z9807 at 0.5, 12, and 48 h, with the highest expression level at 0.5 h. *GhRAF24* is exclusively expressed in Z9807 at 6 h. *GhRAF53*, *GhRAF23*, and *GhRAF14* all showed similar behaviors. The rest of the genes performed poorly in both genotypes. The expression of *GhRAF42* was highest in salt-tolerant cotton lines. These findings suggested that *GhRAF* may also be a significant transcription factor in response to upland cotton genotypes to salt tolerance. As a result, *GhRAF42* gene in the Dt genome of upland cotton for validation, showed upregulated expression. The fact that overexpression is very persistent suggests the importance of salt stress resistance.

### 2.7. Gene Cloning and Subcellular Localization

**Figure 6:**
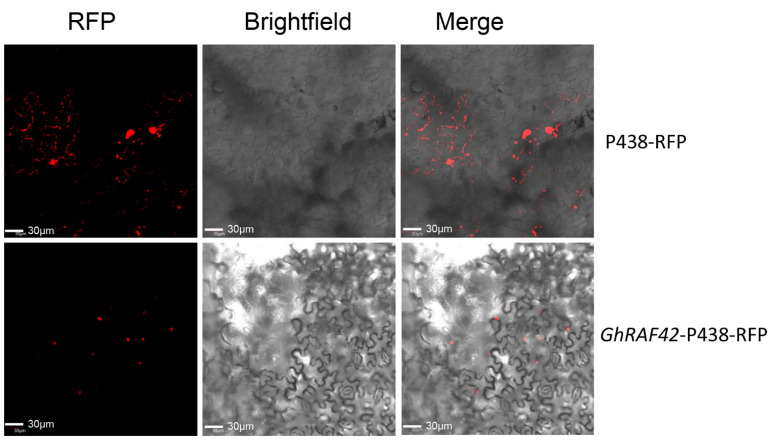
Subcellular location of GhRAF42 proteins. Red fluorescent proteins (RFP)-fusion proteins were transiently expressed in tobacco leaves. After 48 h, florescent genes reveled its presence in nucleus in tobacco leaves. Red represents the expression of RFP gene carried with (without) the GhRAF42 gene, the bar scale is 30 μm.

The target gene *GhRAF42* was cloned using the cDNAs of leaves taken from two cotton genotypes. Agarose gel was used to run the cloned product. The gel imaging equipment detects the size of the cloned target gene to be coherent with the size of the TM-1 reference gene, indicating successful cloning of the target gene. *GhRAF42* has a 4083-bp CDS that encodes 1361 amino acids with an isoelectric point of 5.05 and exhibited a molecular weight of 147.611 kD (Figure S6A). The RAF-like kinase subfamily is predicted by domain analysis. By comparing amino acid sequences of *GhRAF42* gene with DNAMAN10 software, it was possible to find closely homologous sequences of *G. raimondii*, *G. arboreum*, *G. hirsutum*, *G. barbadense,* and *A. thaliana*. Sequence alignment revealed several conserved sequences (Figure S6B). Previously, the GhRAF42 protein sequence was subcellular localization predicted in the nucleus (Table S2). In order to verify the results, the subcellular localization vector GhRAF42-P438-RFP was constructed. Through transient tobacco transformation and image acquisition under laser confocal microscope, it was found that the empty of -RFP was evenly distributed in all parts of the region, while GhRAF42-P438-RFP was only observed on the nucleus (Figure 6).

### 2.8. Silencing of Genes in Upland Cotton and Its Detection

**Figure 7:**
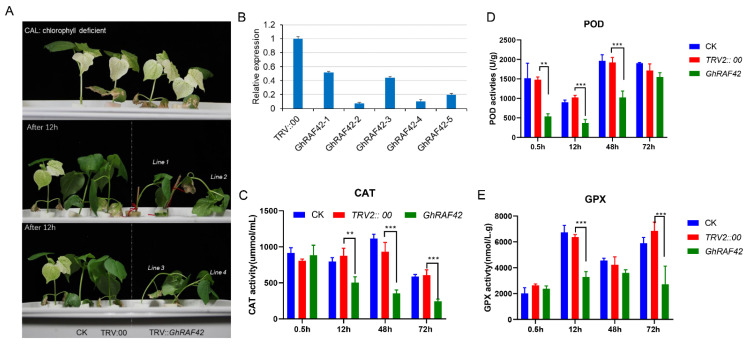
Silencing of GhRAF42 gene by VIGS and enzyme activity (CAT/POD/GPX) analysis under salt stress (**A**) After salt stress, the phenotype of control and gene silenced plants with albino appearance in comparison with CAL: chlorophyll deficient, CK: control, TRV::00: negative control and TRV::*GhRAF42* containing leaves (**B**) Relative expression of 5 cotton plants with silenced gene (**C**) CAT contents in CK: control, TRV2::00 empty vector and TRV2::GhRAF42 candidate gene containing plants. (**D**) POD contents in CK: control, TRV2:00 empty vector and TRV2 = GhRAF42 candidate gene containing plants (**E**) contents in CK: control, TRV2::00 empty vector and TRV2 :: GhRAF42 candidate gene containing plants. The significant differences between transgenic lines and their corresponding control plants is Student ’s *t*-test; ** *p* < 0.01; *** *p* < 0.001.

The *GhRAF42* gene in salt-tolerant cotton variety Z9807 was silenced via VIGS. Genes involved in chlorophyll production were silenced with the insertion of the GhCLA1 gene. As a result, chlorophyll production in cotton was disrupted, and new true leaves developed as an albino in later stages [43]. The albino phenotype develops after infecting the cotton plant, indicating effective gene silencing (Figure 7A). The expression levels of TRV unloaded, *GhRAF42* gene was confirmed by RT-qPCR after the leaves of cotton plants were randomly chosen for fluorescence measurement. Cotton plants normal and unloaded expression levels were unaffected. However, the target gene expression in cotton after silencing decreased considerably, indicating that the *GhRAF42* gene was successfully silenced (Figure 7B). 200 mM salt stress was administered to cotton plants with gene silencing and cotton plants with empty vectors. The phenotype was observed after 12 h of salt stress; gene-silenced cotton plants wilted more severely than control plants (Figure 7A).

To determine candidate gene expression in cotton plants following VIGS silencing. The efficiency of the knockout gene is higher when its expression is low. Figure 7B demonstrates how the expression of knockout genes drops dramatically over time. Five plants were observed at different times, and the knockout gene expression was much lower than the control from expression level 1 to 0.2. TRV2::00 and TRV::*GhRAF42* leaves were collected for RNA extraction and RT-qPCR analysis to investigate the silencing effectiveness of *GhRAF42*. TRV::00 and TRV::*GhRAF42* seedlings were subjected to water for control plants and 200 mM NaCl treatment for 12 h to examine salt tolerance. After the water and NaCl treated TRV2:00 and TRV2:*GhRAF42*, samples were collected from seedlings at 0.5, 12 h, 48 h, and 72 h for quantitative measurements of CAT, POD, and GPX activities.

POD, GPX, and CAT levels were determined using a microplate reader (Figure 6C–E). At 0.5 h after *GhRAF42* gene silencing, there was no significant change in CAT activity between CK and TRV2:00. In the silenced *GhRAF42* seedlings, however, there were highly significant differences between TRV2:00 and *GhRAF42* at 12 h, 48 h, and 72 h. The findings of GPX activity were not the same as those of CAT. The general tendency was upward. At 0.5 h and 48 h after silencing, there were no substantial changes in the *GhRAF42* gene. The content of GPX in cotton plants is much lower than that of CK and TRV:00. TRV2::00 and *GhRAF42* showed highly significant differences at 12 h and 72 h after salt stress treatment. POD activity measurements revealed that the *GhRAF42* gene was significantly different from control and TRV2:00 at 0.5, 12, and 48 h after silencing, and the GPX activity was lower than control and TRV2:00, while control, TRV2::00, and *GhRAF42* showed non-significant expression at 72 h. Overwhelmingly, after *GhRAF42* gene silencing, the CAT, POD, and GPX activities decreased significantly, inferring about the sensitivity of cotton plants against salt stress. 

## 3. Discussion

Raf-like kinases belong to the family MAPKKK, which is phosphorylated by serine/threonine [23]. EDR1 and CTR1 belong to RAF-like kinases and involved in ethylene signal transduction and disease resistance signalling [20,23]. In Arabidopsis, CTR1 is a part of an ethylene receptor signalling complex, which supports a scenario in which CTR1 function requires its localization to the endoplasmic reticulum [44]. EDR1, Raf-like kinase, is also a negative regulator of ethylene-induced senescence and disease resistance [45]. Research also revealed that Raf10 is a new unexplored regulatory component of ABA signaling [46]. RAF is thought to have a function in plant stress response to initiate the diversification process. In this work, one salt-stress-responsive gene of RAF was discovered by transcriptome data filtering.

There were 29, 28, 54, and 56 RAF genes associated with *G. raimondii*, *G. arboreum*, *G. hirsutum* and *G. barbadense*, respectively (Table S2). More than fifty genes exist in both tetraploid species of cotton due to the hybridization of *G. raimondii* and *G. arboreum* occurred about 1–2 Mya, followed by polyploidization. Similar findings have previously been reported in peanut [47] and cotton [48]. According to the comparative analysis of potential RAF genes, gene structure and domain conservation have remained intact throughout the evolution of cotton. The genes that have been discovered, classified into three categories. The conservation of RAF genes was demonstrated throughout evolution by the same group shared by numerous genes in the phylogenetic tree. Similar results were identified in maize [49] and grapevine [50]. The *GhRAF42* gene was shown to be localized in the nucleus according to subcellular localization predictions. IQD gene in soybean, and cotton [51,52] EDR1 in Arabidopsis and tobacco [45,53], CDK and RCC1 gene in cotton [54,55], were also located in the nucleus. While some RAF genes of cotton were located outside the nucleus, in maize, ZmHSF11B2b was also reported in the cytoplasm [56] likewise, PPA gene is detected in cytosol and chloroplast in pear [57].

Generally, segmental duplication was important in the evolution of the RAF gene in cotton. In the current study, purifying the selection of RAF duplicated gene pairs played an essential role in controlling genomic diversity. Moso bamboo [58], Chinese cabbage [59], soybean [60], cotton [61] and grapevine [50] were also undergone purifying selection. Redundant or divergent activities were reported in cotton duplicated genes in response to abiotic stresses [62]. A genome and D genome species have diverged from 2 to 13 Mya [11]. However, transoceanic hybridization of *G. hirsutum* and *G. barbadense* exhibited 1~1.5 mya of A and D genomes [63]. In the same way, duplication experiences in the wheat genome assist the expansion of HSF genes [52] and auxin-responsive GH3 family genes in wheat [64]. Whole-genome duplication of lineage-specific in plant species has acquired a significant number of variations [65]. In the allopolyploid process, gene loss and gene retention increase tolerance against abiotic stresses [66,67,68]. As a consequence, redundant copies of cotton RAF genes might be used to regulate salt tolerance. In cotton, orthologous genes have shown the presence of novel RAF gene subclasses. Similar findings have been reported by Brassica [38].

NaCl was used to treat the RAF-like kinase gene in upland cotton. Salt-sensitive variety was affected more severely by salt stress as compared to salt-tolerant variety. The previous studies have suggested that the RAF18, RAF20, and RAF24 genes played a vital function in salt stress response [69]. Members of the RAF family, such as RAF5 and RAF8, possibly expressed in diverse tissues with increased expression in *A. thaliana*, demonstrating broad response patterns against salt stress [33]. The results revealed that the ability of the CTR1-1 mutant of the RAF-like kinase to resist high salinity is connected to an altered ethylene/auxin regulatory loop in Arabidopsis [70]. Salt stress in cotton causes a disturbance in the dynamic ion balance in plants, changes the average permeability of the membrane, reduces the activity of several membrane-bounded enzymes, and results in a variety of metabolic problems [71]. High salt induces the expression of OsMAPKK1, a distinguished regulator of the salt stress response [72]. Additionally, OsMAPKK6 is implicated in salt and cold stress tolerance [73,74]. Because the two rice MKKs are recognized as mediators of the salt stress response, OsMAPKKK63 might be implicated in the high salinity response as well [75].

Compared to other crops, cotton exhibited higher salt tolerance, yet salt stress significantly impacts cotton development, yield, and quality, particularly in seedling and germination phases. Plant response to salt stress causes changes in gene expression, which leads to alterations in metabolic pathways due to the accumulation of different chemicals [76]. Unnecessary reactive oxygen species are generated in cotton plants during abiotic stress leads to tissue cell damage. An increase in antioxidant enzyme activity was observed in the cell upon oxidizing a tissue, eliminating excess active oxygen and protecting plants from damage caused by unfavorable stress. As a result, the activity of antioxidant enzymes in plants may be used to measure the stress tolerance of a plant [77].

The appearance of an albino phenotype in plants confirmed the silencing of candidate gene *GhRAF42* (Figure 6). The amount of chlorophyll in cotton leaves provides a straightforward and accurate measure for assessing the cellular damage induced by salt stress [78,79]. Furthermore, after silencing the targeted gene, its expression was considerably decreased. After salt stress treatment, the silenced cotton plants showed significant wilting as well. *GhRAF42* gene silencing suggested that it protects cotton plants from salt stress. Likewise, improved salt tolerance was reported by downregulation of *GhNHX1*, *GhWRKY6,* and *RCC1* in VIGS-treated cotton [59,80,81]. After gene silencing, activity of CAT, GPX, and POD in cotton plants was much lower than that of non-VIGS plants at various time intervals. Infact, silencing the *GhRAF42* gene decrease the ability of cotton plant to repair the damage produced by reactive oxygen species, which makes them more sensitive to salt stress. In cotton, It was found that salinity stress reduced the CAT, GPX, and POD content in leaves [71]. Overwhelmingly, quantitative fluorescence experiment, VIGS, and exposure of physiological signs after silencing reflect that the *GhRAF42* gene plays a significant role in salt tolerance in cotton plants.

## 4. Material and Methods

### 4.1. Classification and Characterization of RAF Proteins in Cotton

Alignment of *G. raimondii* (JGI), *G. arboreum* (CRI), *G. hirsutum* (CRI), and *G. barbadense* (HAU) genomes sequences were downloaded from the cotton functional genomics database (CottonFGD; https://cottonfgd.org/about/download.html, accessed on 23 June 2021). The RAF protein sequences were achieved from *A. thaliana* using software HMMER (v3.3.2) with 1e^−10^ of e value [82], and the targeted candidate genes were analyzed using the program BLASTP [83]. NCBI CDD search (http://www.ncbi.nlm.nih.gov/cdd/, accessed on 23 June 2021) and Pfam (http://pfam.xfam.org/scan, accessed on 24 June 2021) were also used to confirm candidate protein sequences (Pfam ID: PF07714). The physiochemical properties of RAF proteins were determined using Expert Protein Analysis System (ExPASy) (http://www.expasy.org/, accessed on 27 June 2021). Whereas, Bologna Unified Subcellular Component Annotator (http://busca.biocomp.unibo.it, accessed on 03 July 2021) was utilized to predict the subcellular localization [84,85]. Gr, Ga, Gb and Gh prefixes were used for *G.* *raimondii*, *G. arboreum*, *G. hirsutum*, and *G. barbadense,* respectively.

### 4.2. Multiple Sequence Alignment and Intron or Exon Structure Analysis of RAF Gene Family

Clustal Omega, (https://www.ebi.ac.uk/Tools/msa/clustalo/, accessed on 07 July 2021) was used to align the RAF gene sequences of four cotton species by using default parameters [86]. To discover conserved motifs in RAF proteins, MEME (Multiple Em for Motif Elicitation) version 5.4.1 [87] was utilized, and the settings were optimized with a total of 12 motifs, at least 5 motifs per protein and motif width was 25 to 200 bp [88]. Coding regions and genomic sequences of cotton were examined to determine the structure of genes. In addition, the pattern distribution and splicing process of introns were revealed using aligned sequences of CDS. The intron/exon arrangement of RAF genes was elucidated using TBtools (v1.098661) [89]. 

### 4.3. Phylogenetic Analysis of RAF Genes and Gene Ontology Analysis

The evolutionary tree was generated with the help of Molecular Evolutionary Genetics Analysis (MEGA (vX)) by using neighbor-joining method, which included 28 RAF genes from *G. raimondii*, 29 from *G. arboreum*, 56 from *G. hirsutum*, 54 from *G. barbadense*, and 22 from *A. thaliana* [39]. Tree nodes were calculated using the bootstrap method with 1000 replications for statistical strength [90]. At the same time, evolutionary distances were calculated by the number of differences and amino acid as substitution type with uniform rates and patterns [91]. Meanwhile, gene ontology (GO) enrichment analyses were performed using the CottonFGD database (accessed on 11 September 2021).

### 4.4. Chromosomal Location, Synteny Analysis, and Collinearity Analysis of RAF Genes

Chromosomal locations of the RAF genes in four cotton species were determined using CottonFGD genome annotation data (accessed on 25 June 2021). Each RAF gene was mapped and displayed on each chromosome by using Mapchart (v2.32) [92]. MCScanX (v0.8) [93] was used to identify gene duplication via TBtools [89]. Collinearity maps were created using TBtools with circos and synteny programs to illustrate segmentally duplicated and orthologous pairs of RAF genes between and within cotton genomes. The nonsynonymous (Ka) and synonymous (Ks) substitution values of RAF genes were computed through TBtools. When the Ka/Ks = 1, it indicates neutral selection; when greater than 1, it suggests positive selection; and less than 1 specifies purifying selection [94,95]. Formula T = Ks/2λ × 10^−6^ (Mya) was used to compute duplicated events, whereas λ = 1.5 × 10^−8^ in cotton [96].

### 4.5. Identification of Orthologous RAF Genes Based on Sequence 

OrthoVenn2 (https://orthovenn2.bioinfotoolkits.net/home, accessed on 02 July 2021) was used with default settings to identify orthologous genes in *G. raimondii*, *G. arboreum*, *G. hirsutum* and *G. barbadense* [97]. The study included the sequences of known RAFs proteins from all four cotton species and protein sequences from *A. thaliana*. Furthermore, each cotton species was assessed in each possible combination of cotton and *A. thaliana* to discover potential orthologous gene clusters.

### 4.6. Heat-Map Analysis 

The expression levels of RAF genes were determined using fragments per kilobase per million (FPKM) values from the ”TM-1” cultivar transcriptome data obtained from the NCBI database (https://www.ncbi.nlm.nih.gov/bioproject/?term=prjna248163, accessed on 14 May 2021). Trimomatic software (v0.38) was used to remove adaptors to conduct quality control [98]. Hisat2 software was used to map the genome reads, and Cufflinks was used to get the consistent FPKM values [99,100]. The results of RAF genes were log-transformed operating threshold standards established on the false discovery rate (FDR) statistical technique and the FPKM ratio, i.e., FDR less than 0.001 and |log2 Ratio| ≥ 1. Using TBtools, heatmap was created to illustrate the RAF family gene expression [89]. 

### 4.7. Plant Materials

One salt-tolerant cotton genotype, Zhong9807 (Z9807), and one salt-sensitive variety, Zhong J0102 (ZJ0102), were collected from Cotton Research Institute (CRI), Chinese Academy of Agricultural Sciences (CAAS), Anyang, China. Cotton seeds were de-linted and germinated for three days at 25 °C on wet filter paper before being transferred to hydroponic pots with hoagland nutrient solution [101] in a greenhouse with 60–70% humidity, 14 h photoperiod, and 28 °C day/night temperature. On the emergence of 3rd true leaf, the seedlings were subjected to salt stress with 100 mM, 150 mM, and 200 mM sodium chloride (NaCl) solutions. Plants in the control group did not receive any salt treatment. Each treatment was repeated three times. After0-(CK) 0.5-, 3-, 6-, 12-, 24-, and 48 h five individual seedling leaves were collected for each biological replication and immediately put in liquid nitrogen and kept at −80 °C.

### 4.8. RNA Extraction from Upland Cotton for Real-Time Quantitative PCR (RT-qPCR) Analysis

According to the manufacturer’s protocols, RNA was isolated from root and leaf samples using an RNA extraction kit, the RNA-prep Pure Plant kit (Tiangen, Beijing, China). NanoDrop 2000 spectrophotometer (Thermo Scientific, Wilmington, DE, USA) and gel electrophoresis (Biobase, Jinan, China) were used to examine RNA sample concentration and its consistency. Only RNA with 260/280 ratio of 1.8–2.1 and a 260/230 ratio of 2.0 was stored at −80 °C and used for further studies. As an internal control, Actin was used to normalize cDNA amplification in each reaction, and specific gene primers, i.e., *GhRAF42* were used for RT-qPCR (Table S1). The 1st Strand cDNA Synthesis SuperMix kit (NO. E047-01B; Novoprotein, Shanghai, China) was used in RT-qPCR to synthesis the cDNA first strand according to the manufacturer’s protocols. Primer Premier5 developed particular primers for the RAF gene with melting temperatures of 55 to 60 °C, primer lengths of 18 to 25 base pairs, and amplicon lengths of 101 to 221 bp. RT-qPCR was performed using Universal SYBR Quick Start Green Master according to manufacturer recommendations (Rox) (Roche, Mannheim, Germany). The total master mix was 20 µL, with 2 µL of cDNA template, 2 µL of each primer, 6 µL of deionized H_2_O, and 10 µL of SYBR green master mix (Novostar@ SYBR qPCR SuperMix Plus (Code NO. E096-01A; Novoprotein, Shanghai, China)). The PCR thermal cycling conditions were as follows: 95 °C for 10 min, followed by 40 cycles of 95 °C for 5 s, 60 °C for 30 s, and 72 °C for 30 s. The data was recorded at 95 °C for 15 s, 60 °C for 1 min, 95 °C for 30 s, and 60 °C for 15 s during the extension process. Three biological and technical replications were performed on each cDNA sample. GhActin (GI: AY305733) was used as an internal standard to standardize cDNA content in this study. The data was processed using the 2^−ΔΔCt^ method [102], and the heatmap was created with the TBtools program.

### 4.9. Subcellular Localization

Primer Premier (v5.0) was used to construct specific primers based on the CDS sequence of the *GhRAF42* genes (Table S1). To develop the fusion structure of translation RFP, the CDS and restriction site of insertion into the SalI restriction site of the pBinRFP vector were evaluated. According to the methodology, the recombinant plasmid was transformed into Agrobacterium tumefaciens strain LBA4404 then inserted into the second or third leaf of the tobacco. Under the same conditions, the vector of pBinRFP (RFP alone) was transformed into tobacco for planting control. Finally, the diseased tobacco leaves were covered in tin foil and placed in the dark for 48 h. After 48 h, use scissors to trim the leaves enclosing the injection site carefully. The altered gene expression was examined by CCD optical microscope observation (Leica Microsystems, Wetzlar, Germany).

### 4.10. Virus-Induced Gene Silencing (VIGS) in Cotton

As previously reported by Bachan et al. [103], the Tobacco rattle virus (TRV) system was used to analyze VIGS. A 300-bp fragment of the RAF gene was cloned using BamH1 and Xba1 restriction sites into the pTRV-RNA vector to create TRV::*GhRAF42*. Primer Premier (v5.0) software was used to generate primers (Table S1). TRV::GhCLA1 was also designed as a visual marker to track silencing effectiveness. Empty vector TRV2::00 was utilized as a negative control. All vectors were introduced into the LBA4404 strain of *Agrobacterium tumefaciens*. 10-days old cotyledons of *G. hirsutum* of Z9807 seedlings were injected at a temperature of 25 °C. After 2 weeks of infiltration, the lines were injected with TRV::GhCLA1, resulting in an albino phenotype. Salt treatment of 200 mM was started on the emergence of three leaves stage until phenotype appeared, while control plants were watered with 1/2 MS nutrient solution.

### 4.11. Assessment of Enzyme Activity Content of Gene Silencing Cotton

Wild-type seedlings and transgenic cotton seedlings were treated with 200 mM NaCl solution after four weeks. The concentration of glutathione peroxidase (GPX), peroxidase (POD), and catalase (CAT) in leaves was measured at various time intervals following salt treatment. After 0.5, 12 h, 48 h, and 72 h, five individual seedling leaves from each biological replication were collected, and examined for enzyme content. Control pots were irrigated with tap water. Weigh each centrifuge tube, add roughly 0.1 g leaves to each tube, and separate the treated cotton. The protein content of the crude leaf extract was measured before the enzyme activity content. Three separate biological replicates of each control and salt-treated sample (3–4 seedlings) were assessed. The CAT, POD and GPX activities were determined using a CAT Assay Kit (A007-1), POD Assay Kit (A084-3), and GPX Assay Kit (A004-3) according to the protocol manufactured by Nanjing Jiancheng Bioengineering Institute, Nanjing, China.

## 5. Conclusions

Salt stress is a major issue that harms the plant production and growth all around the globe. *GhRAF42* gene was used to assess the involvement of RAF-like kinases in plant stress tolerance. From transcriptome data, 56 genes were identified, with differential gene expression under salt stress. Salt tolerant Z9807 and salt-sensitive ZJ0102 genotypes were subjected to salt stress, and potential genes were identified. Collinearity and synteny analysis revealed the role of segmental duplications in the evolution of RAFs in cotton. Candidate genes are found in the nucleus, as predicted by subcellular localization. Target gene silencing caused evident wilting under 200 mM NaCl treatment. Control plants and plants treated with negative genes had decreased GPX, POD and CAT levels, suggesting that the *GhRAF42* gene is linked to salt tolerance. This research might help scientists to understand better the role of RAF-like kinases in plants to identify possible genes for developing salt resistance in cotton genotypes.

## Supplementary Materials

The following are available online at https://www.mdpi.com/article/10.3390/ijms222312649/s1.
