# Supplementary material for: Identification of Raf-Like Kinases B Subfamily Genes in Gossypium Species Revealed GhRAF42 Enhanced Salt Tolerance in Cotton
Source: Int J Mol Sci. 2021 Nov 23;22(23):12649. doi: 10.3390/ijms222312649 (PMC8657469; doi:10.3390/ijms222312649)
Supplement: Supplementary file 1 [file ijms-22-12649-s001.zip › Supplementary Figure S1-S6.pdf]

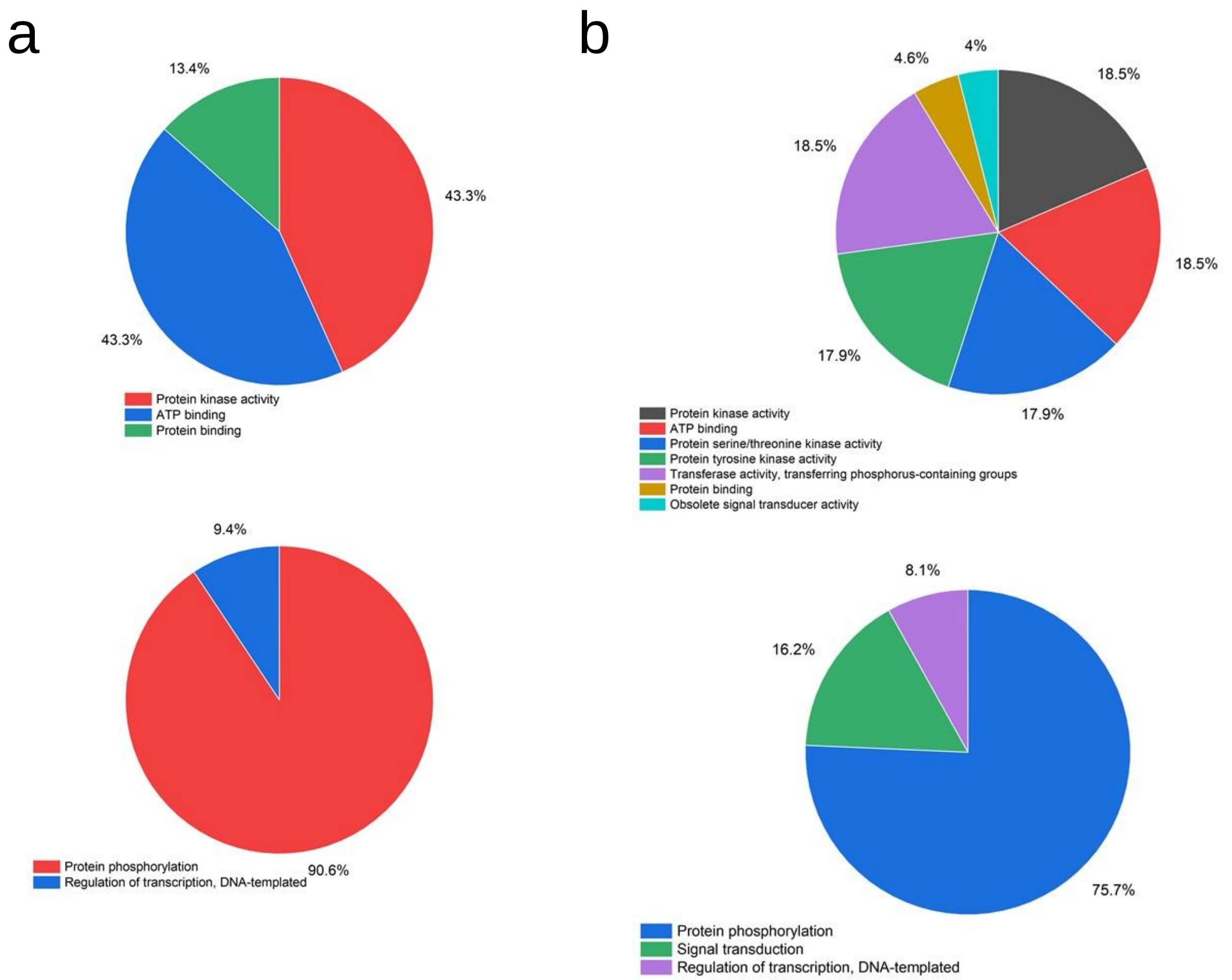

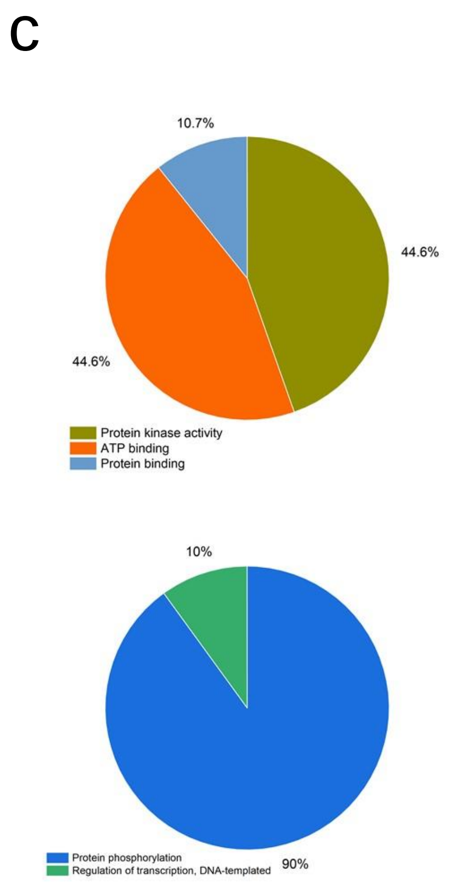

Figure S1. Analysis of RAF gene ontology to study molecular and biological functions (A) *G. arboreum* (B) *G. raimondii* (C) *G. barbadense*:

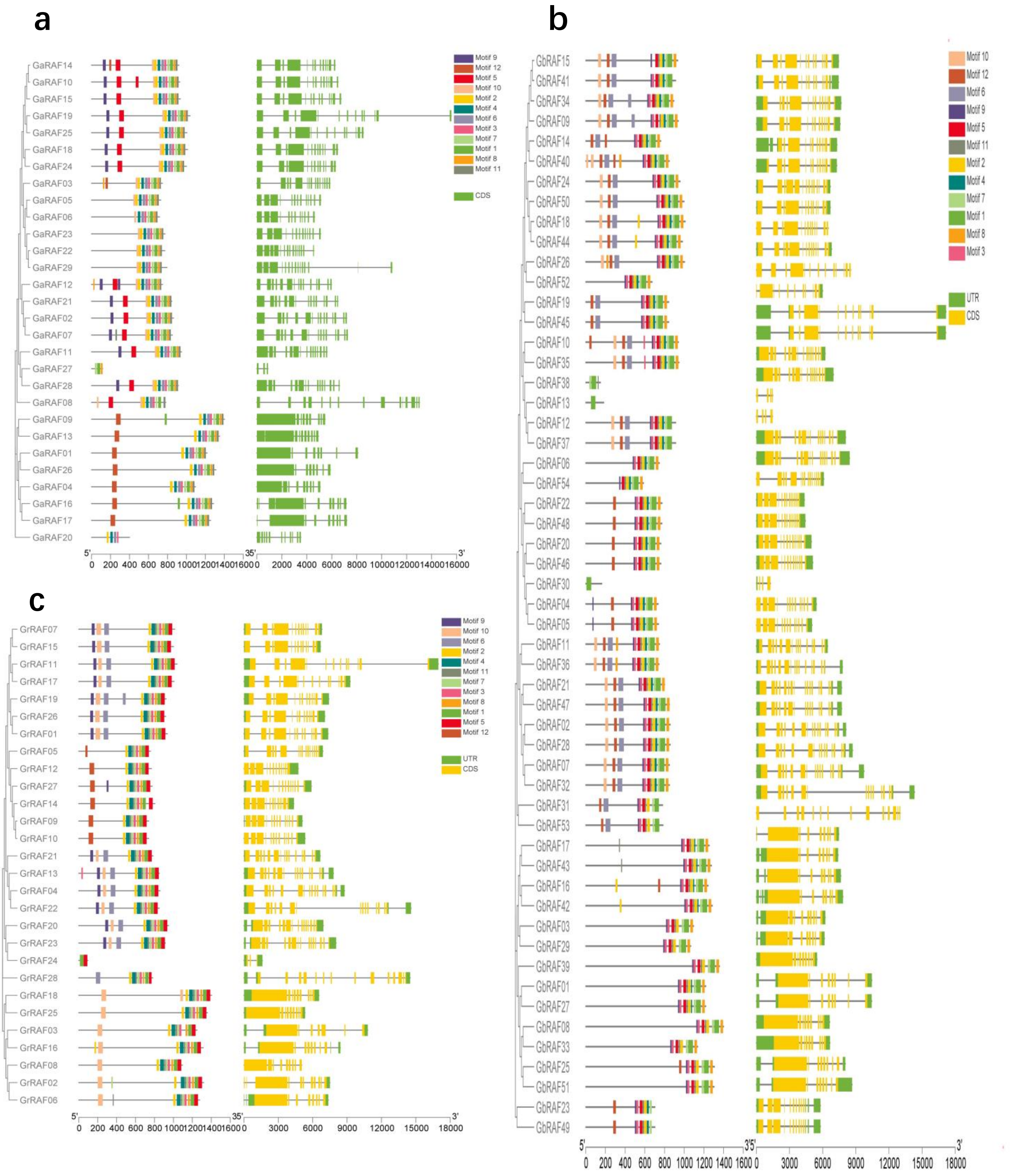

Figure S2: Phylogenetic tree, Motif and Gene structure of RAF gene Family of cotton (A) *G. arboreum* (b) *G. barbadense* (c) *G. raimondii*

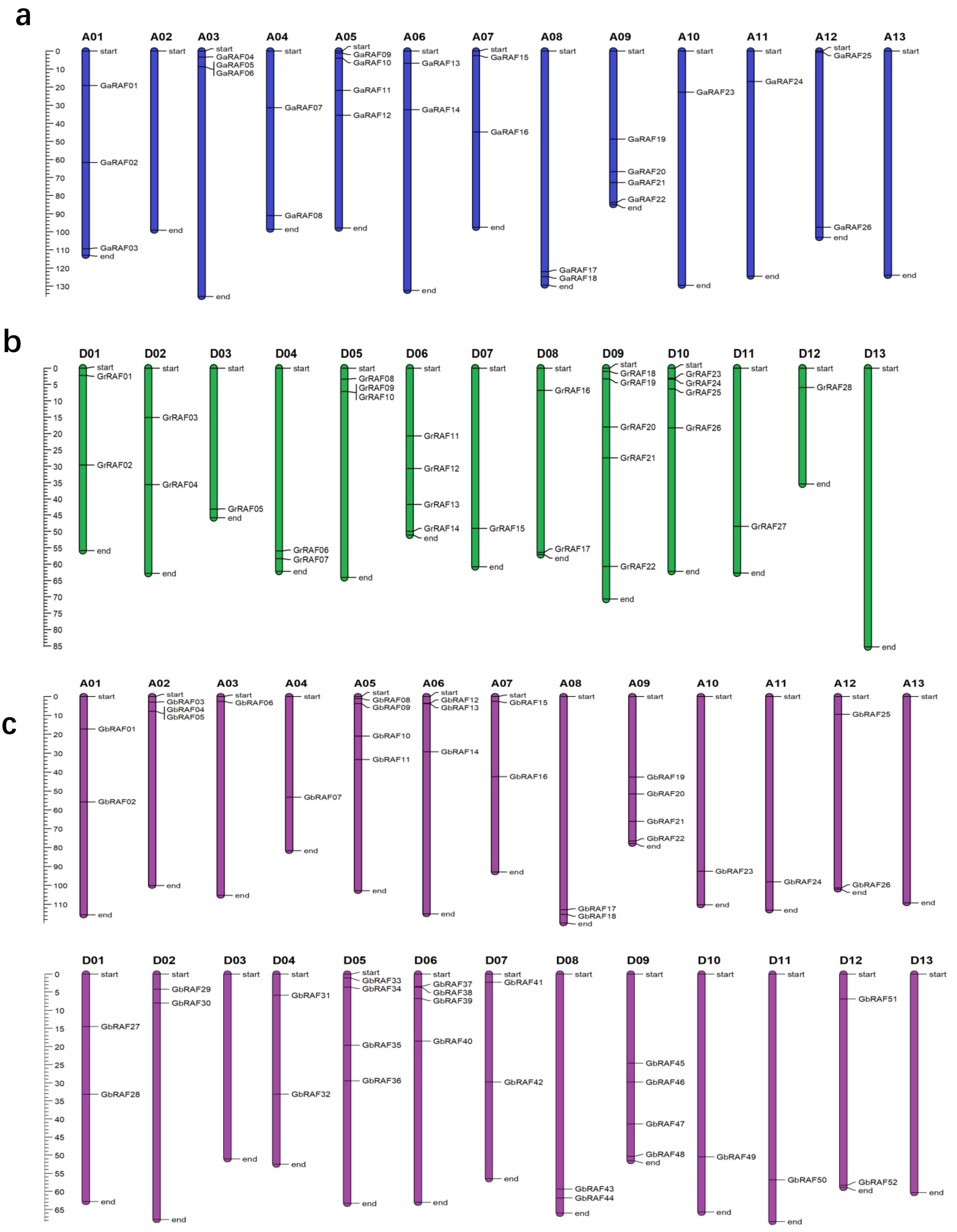

Figure S3. Chromosomal map of RAF gene family of cotton (A) *G. arboreum* (B) *G. raimondii* (c) *G. barbadense*

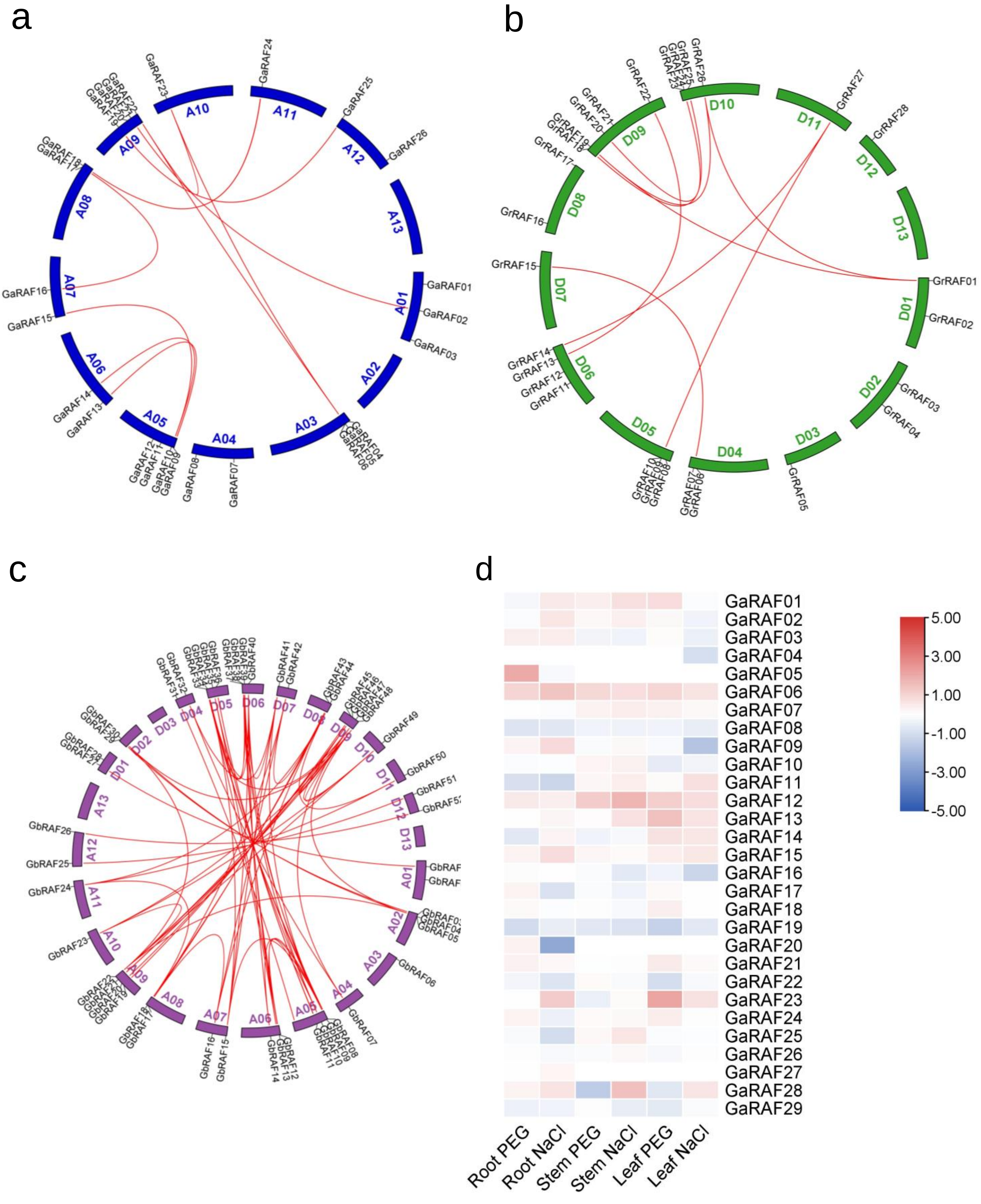

Figure S4: Collinearity and expression analysis of RAF gene family in cotton (a) *G. arboreum* (b) *G. raimondii* (c) *G. barbadense* (d) Expression analysis of *G. arboreum*

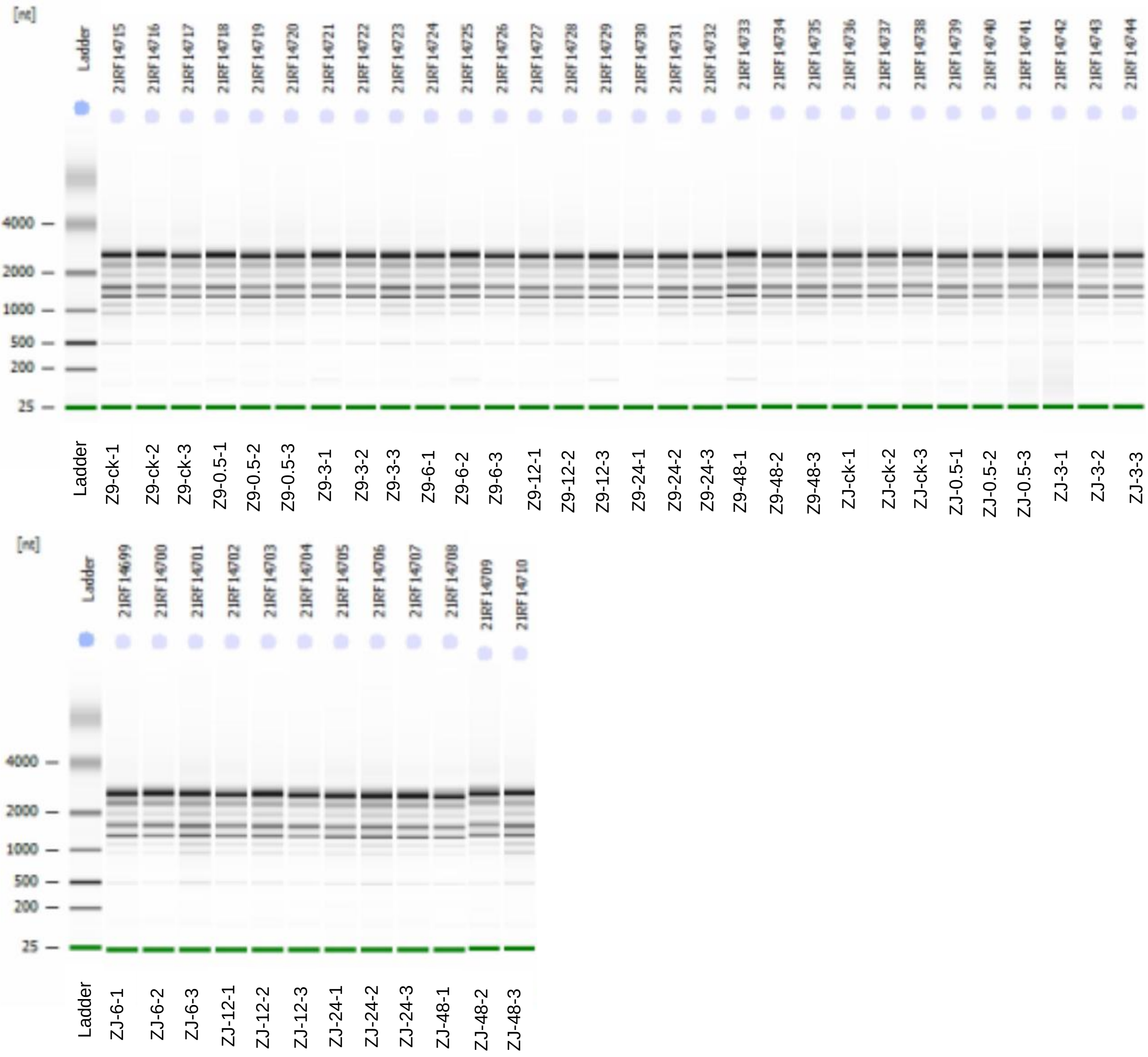

Figure S5: Analysis of the quantity, integrity and purity of total RNA for 42 leaf samples with the Agilent 2100 Bioanalyzer for qRT-PCR and RNA-seq. CK means NaCl-untreated as control.0.5h-48h means different time points after NaCl stress. Z9:Z9807;ZJ:ZJ0102.

a

b

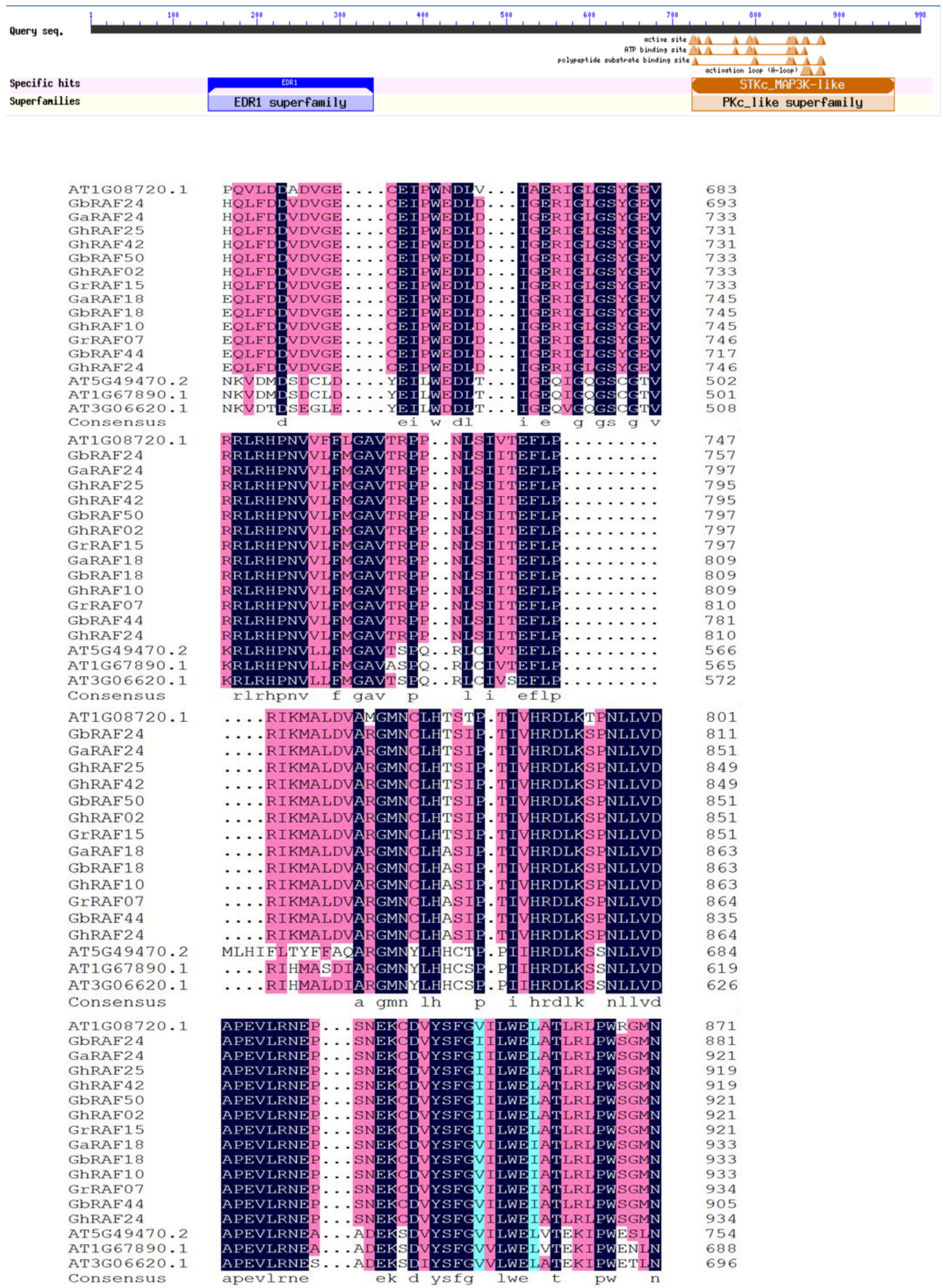

Figure S6: *GhRAF* gene cloning, conserved domain database and sequence alignment analysis (A) Conserved domain database analysis of GhRAF42 (E) Multiple sequence alignment of GhRAF42
